# Influencing factors of lymphopenia after conventional fractionated whole-breast radiotherapy following breast-conserving surgery for early breast cancer: a retrospective study

**DOI:** 10.3389/fonc.2025.1669347

**Published:** 2026-01-09

**Authors:** Yang Sun, Yanan Chen, Ting Liu, Pan Pan, Yongjuan Gu, Xuexue Liang, Dong Shen, Lixian Xu

**Affiliations:** 1 Department of Oncology, The First People’s Hospital of Zhangjiagang City, Soochow, China; 2 Linear Accelerator Center, The First People’s Hospital of Zhangjiagang, Soochow, China

**Keywords:** breast tumor, conventional whole-breast radiotherapy, dosimetry, lymphopenia, sternum

## Abstract

**Objective:**

To explore the risk factors for grade 2 or higher lymphocytopenia during whole-breast radiotherapy after breast-conserving surgery for breast cancer.

**Methods:**

A total of 112 patients who underwent whole-breast radiotherapy after breast-conserving surgery for breast cancer in our department from June 2019 to June 2024 were selected and divided into the no/mild lymphopenia group (no or grade 1 lymphocytopenia, 48 cases) and moderate/severe lymphopenia group (grade 2 or higher lymphocytopenia, 64 cases). Univariate and multivariate logistic regression analyses were performed on the clinical data and dosimetric parameters of the two groups to identify the independent risk factors for grade 2 or higher lymphocytopenia. The predictive efficacy of the risk factors was established using the receiver operating characteristic (ROC) curve.

**Results:**

Univariate logistic regression analysis indicated that there were statistically significant differences between the two groups in terms of whether they received chemotherapy, the quantity of chemotherapy cycles, initial lymphocyte count prior to chemotherapy and radiotherapy, and dosimetric parameters, such as the mean dose (Dmean), doses received by 40%, 60%, and 80% of the sternum volume (D40, D60, D80), and volume of the sternum receiving 5 Gy (V5) (OR range: 0.092~3.927, *P* < 0.05). Multivariate logistic regression analysis revealed that lymphocytopenia prior to chemotherapy and an elevated D80 of the sternum were independent predictors for grade 2 or higher lymphocytopenia (OR range: 0.287~1.517, *P* < 0.05). The ROC curve analysis determined that the optimal threshold values for initial lymphocyte count prior to chemotherapy and D80 of the sternum were 1.775 × 10^9^/L and 2.85 Gy, respectively. The area under the curve (AUC) values were 0.787 and 0.7, with corresponding sensitivities of 82.8% and 75.6%, and specificities of 70.4% and 76.7%, respectively.

**Conclusion:**

Lymphocytes <1.775 × 10^9^/L before chemotherapy and D80 of the sternum >2.85 Gy are independent risk factors for grade 2 or higher lymphocytopenia during whole-breast radiotherapy after breast-conserving surgery for breast cancer.

## Introduction

Breast cancer ranks among the most prevalent types of malignant neoplasms affecting women. For individuals diagnosed with early-stage breast cancer who have received breast-conserving surgery, the standard therapeutic approach involves the administration of radiotherapy to the entire breast and the tumor bed. This approach can reduce the risk of recurrence, metastasis, and mortality while also improving survival benefits (1). Nevertheless, it inherently subjects the sternum and the affected ribs to radiation exposure, which has the potential to harm hematopoietic cells. Additionally, chemotherapy can affect bone marrow proliferation, leading to a higher incidence of hematological toxicity during radiotherapy. Previous studies had primarily focused on changes in indicators such as white blood cells and neutrophils. Recent research has revealed that lymphocytopenia during radiotherapy is associated with fatigue and can negatively impact patient outcomes in the long term (2, 3). Consequently, conducting an investigation into the factors that contribute to the development of grade 2 or higher lymphocytopenia subsequent to postoperative radiotherapy for breast cancer and instituting preventative measures hold significant clinical importance (4). This study retrospectively analyzed the clinical data and dosimetric parameters of 112 patients who underwent postoperative radiotherapy at our department from June 2019 to June 2024. The aim was to identify the risk factors for grade 2 or higher lymphocytopenia, providing a clinical reference for whether postoperative adjuvant radiotherapy for breast cancer patients leads to lymphocytopenia.

## Materials and methods

### Study subjects

A retrospective analysis was performed on 134 individuals diagnosed with breast cancer who underwent postoperative radiotherapy subsequent to breast-conserving surgery at the First People’s Hospital of Zhangjiagang City between June 2019 and June 2024. The inclusion criteria were as follows: 1) age between 18 and 80 years, 2) histopathological confirmation of breast cancer, 3) TNM staging of TisN0M0 or T1-2N0-1M0, and 4) receipt of standard whole-breast synchronous or sequential local dose escalation radiotherapy. The exclusion criteria encompassed 1) bilateral breast cancer; 2) prior chest radiotherapy, incomplete radiotherapy, or radiotherapy in the supraclavicular, infracostal, or upper and lower mediastinal lymph node drainage areas; 3) immune system diseases or severe cardiopulmonary dysfunction; and 4) incomplete or missing clinical data. A total of 22 individuals were excluded from the analysis, comprising 15 with missing or incomplete data; 1 with stage N2; 5 with supraclavicular, infracostal, or upper and lower mediastinal irradiation; and 1 with incomplete radiotherapy. Consequently, the study population consisted of 112 individuals. The study was approved by the Ethics Committee of the First People’s Hospital of Zhangjiagang City (Approval Number: 25GYYLL‐2025‐05-055), and the exemption from signing an informed consent form was granted for the retrospective study.

### Radiotherapy *target area p*lanning and *p*rotocol *for the whole breast and tumor bed a*rea

All patients underwent routine whole-breast radiotherapy with synchronous or sequential dose escalation to the tumor bed area. The target area planning for the whole breast and tumor bed area followed the European Radiation Therapy Oncology Group (RTOG) guidelines for post-radical mastectomy target area planning (5). The radiotherapy plan is as follows: For the simultaneous integrated boost (SIB), the total radiation dose for the entire breast is 45–50 Gy, divided into 25 fractions, with 1.8–2.0 Gy per fraction, 5 times per week. The tumor bed will receive a synchronous dose escalation to 60 Gy, 2.4 Gy per fraction, for a total of 5 weeks. For the sequential boost (SB), the total radiation dose for the entire breast is 50 Gy, divided into 25 fractions, 2.0 Gy per fraction, 5 times per week. The tumor bed will receive a sequential dose escalation to 60 Gy, 2.0 Gy per fraction, for a total of 6 weeks. The prescription dose covers 95% of the PTV volume, and all critical organs meet the normal tissue limit.

### Bone marrow delineation

The sternum (including the sternal handle, body, and xiphoid process) and the affected ribs (upper boundary from the upper edge of the first rib to 3.0 cm below the entire breast target area) are delineated, with the external contour of the bone used to represent the total irradiated bone marrow. Based on the treatment plan, the corresponding dose–volume histogram (DVH) is derived, recording the volume of the entire breast (Dmean) and the volume of the sternum and ribs [V5, V10, V20, V30, V40, Dmean, D20, D40, D60, and D80, where Dmean is the mean dose received, Vx is the volume receiving ≥x Gy, and Dx is the volume receiving x % of the dose (volume unit: cm³; dose unit: Gy)].

### Observation indicators

Peripheral lymphocyte count (PLC) was measured pre-chemotherapy, during chemotherapy, pre-radiotherapy, during radiotherapy, and 1–2 weeks after radiotherapy, with the patients’ blood counts routinely tested to record the PLC. The lymphocytopenia grading was based on the Common Terminology Criteria for Adverse Events (CTCAE) 5.0, which is as follows: grade 0 (PLC ≥ normal lower limit), grade 1 (0.8 × 10^9^/L ≤ PLC < normal lower limit), grade 2 (0.5 × 10^9^/L ≤ PLC < 0.8 × 10^9^/L), grade 3 (0.2 × 10^9^/L ≤ PLC < 0.5 × 10^9^/L), and grade 4 (PLC < 0.2 × 10^9^/L). In the present study, the normal lower threshold for PLC is 1.1 × 10^9^/L.

### Statistical analysis

Data were analyzed using SPSS 22.0 software. For continuous variables that meet the conditions of normal distribution and homogeneity of variance, *t*-tests were used for comparisons between groups, with data expressed as $\bar{\text{x}}\pm \text{s}$. For continuous variables that do not meet the conditions for *t*-tests, the Wilcoxon rank sum tests were used, with data expressed as *M* (IQR). Categorical data were presented as cases (%), and intergroup comparisons were conducted using *χ*^2^ tests or Fisher’s exact probability method. Univariate and multivariate logistic analyses were used to explore the factors associated with grade 2 or higher lymphocytopenia during radiotherapy. The difference was statistically significant when *P* < 0.05.

## Results

### The *relationship b*etween PCL *reduction and r*adiotherapy

**Figure 1:**
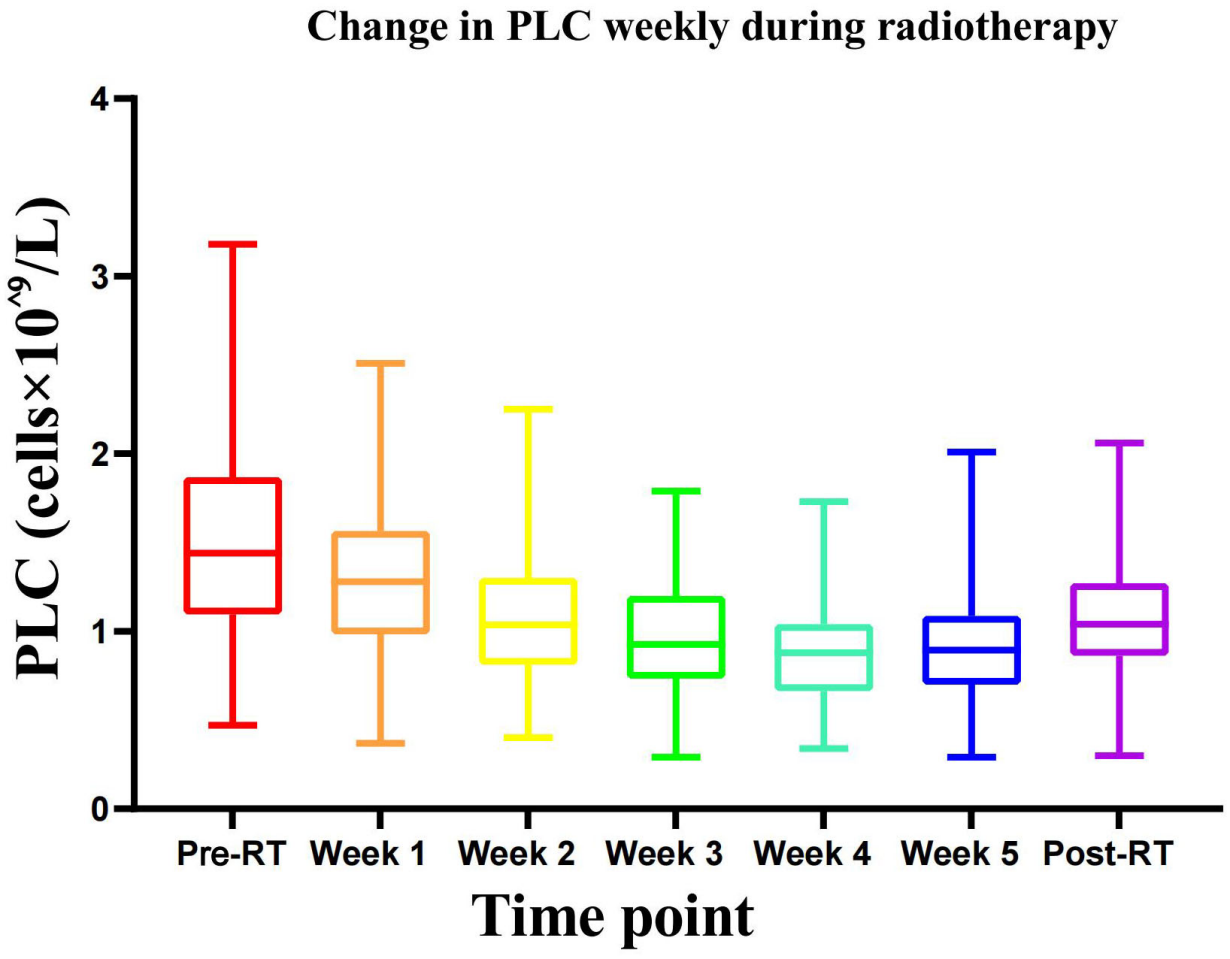
Change in PLC weekly during radiotherapy.

As shown in **Table 1**; **Figure 1**, the PLC levels decreased within the first week of radiotherapy. Subsequently, the reduction persisted in diminishing with the aggregate dose of radiotherapy, attaining its nadir by the fourth week and experiencing a gradual recovery thereafter. Prior to the commencement of radiotherapy, reductions in PLC levels of 0–3 were observed in 84, 17, 11, and 1 patients, respectively. Throughout the entire radiotherapy period, 13, 35, 47, and 17 patients experienced 0–3 level PLC reductions, with no 4–5 level adverse reactions. The 0–1 level was designated as the no/mild lymphopenia group (N/MLG) (48 cases), and the 2–3 level was defined as the moderate/severe lymphopenia group (M/SLG) (64 cases). After treatment, the mean PCL levels of both groups were significantly lower than pretreatment levels (*P* < 0.05). Apart from the minimum PLC values during chemotherapy, which showed no significant difference between the two groups, all the other aspects were statistically significant (*t* range: 3.625~13.8, *Z* = −4.298, *P* < 0.05).

**Table 1:** Comparison of lymphocyte counts in patients with radiotherapy after breast-conserving surgery for breast cancer [( $\bar{\text{x}}\pm s$) or *M* (IQR)] (cells × 10^9^/L).

Item	*N* (N1/N2)	N/MLG	M/SLG	Statistic	*P*	*N* (N1+N2)
Pre-CT	29/54	2.01 (0.59)	1.57 (0.71)	*Z* = −4.298	<**0.001**	1.82 ± 0.64
Lowest during CT	29/54	0.69 (0.52)	0.62 (0.36)	*Z* = −1.314	0.189	0.69 ± 0.35
Pre-RT	48/64	1.84 ± 0.51	1.24 ± 0.50	*t* = 6.245	<**0.001**	1.50 ± 0.58
Week 1	48/64	1.62 ± 0.39	1.05 ± 0.30	*t* = 8.401	<**0.001**	1.30 ± 0.44
Week 2	48/64	1.41 ± 0.38	0.87 ± 0.22	*t* = 8.771	<**0.001**	1.10 ± 0.40
Week 3	48/64	1.21 ± 0.22	0.78 ± 0.20	*t* = 10.583	<**0.001**	0.96 ± 0.30
Week 4	48/64	1.15 ± 0.23	0.7 ± 0.17	*t* = 11.344	<**0.001**	0.89 ± 0.30
Week 5	48/64	1.14 ± 0.29	0.77 ± 0.29	*t* = 6.625	<**0.001**	0.93 ± 0.34
Pro-RT	48/64	1.22 ± 0.32	1.0 ± 0.33	*t* = 3.625	<**0.001**	1.09 ± 0.34
Lowest during RT	48/64	1.01 ± 0.18	0.60 ± 0.13	*t* = 13.8	<**0.001**	0.78 ± 0.25

N/MLG, No/Mild Lymphopenia group; M/SLG, Moderate/Severe Lymphopenia Group; Pre-RT, before radiotherapy; Week1-Week5, from the first to the fifth week of radiotherapy; Post-RT, after radiotherapy.The bolded values indicate that the figures have statistical significance (P < 0.05)

### Comparison of clinical data between the two groups of patients

A total of 112 patients diagnosed with breast cancer, with a mean age of 53.3 ± 10.85 years, were examined. Statistical analysis revealed no significant differences in age, surgical approach, molecular subtype, T/N stage, tumor location/quadrant, estrogen receptor/progesterone receptor status, human epidermal growth factor receptor 2 status, histological grade, the interval between radiotherapy and chemotherapy, and the method of dose escalation in the tumor bed among the patients (*χ*^2^ range: −0.917~4.511, *t* = 0.088, *Z* = −0.262, *P* > 0.05), as delineated in **Table 2**. Of the 112 patients, 29 did not undergo chemotherapy prior to radiotherapy, 11 were administered a regimen of anthracycline and cyclophosphamide, 30 received a combination of anthracycline and cyclophosphamide followed by paclitaxel, and 42 were treated with chemotherapy protocols that included taxane drugs, with or without the addition of cyclophosphamide or carboplatin. Chemotherapy regimens and the increase in the number of chemotherapy cycles were significantly associated with a reduction in grade 2 or higher PCL (*P* < 0.05). Compared to the N/MLG, the proportion of patients with more than 4 chemotherapy cycles was higher in the M/SLG (48.5% *vs*. 31.2%).

**Table 2:** Baseline characteristics of 112 patients with breast cancer who underwent breast-conserving surgery before radiotherapy [*N* (%) or $\bar{\text{x}}\pm s$ or *M* (IQR)].

Characteristics	N/MLG (48)	M/SLG (64)	Statistic	*P*	Characteristics	N/MLG (48)	M/SLG (64)	Statistic	*P*
Age (years)	53.41 ± 10.84	53.23 ± 10.95	*t* = 0.088	0.93	Quadrant			*χ*^2^ = 4.049	0.263
Preservation			*χ*^2^ = 3.764	0.052	Inside-up	8 (16.7)	18 (10.9)		
Sentinel LN-bio	46 (95.8)	54 (84.4)			Inside-down	8 (16.7)	7 (48.4)		
Armpit cleaning	2 (4.2)	10 (15.6)			Outside-up	29 (60.4)	31 (12.5)		
T			*χ*^2^ = 2.853	0.24	Outside-down	3 (6.2)	8 (42.2)		
Tis	8 (16.7)	5 (7.8)			Histological			*χ*^2^ = 0.176	0.916
T1	31 (64.6)	41 (64.1)			I–II	24 (50)	30 (46.9)		
T2	9 (18.7)	18 (28.1)			III	14 (29.2)	21 (32.8)		
N			*χ*^2^ = 1.854	0.173	Others	10 (20.8)	13 (20.3)		
N0	44 (91.7)	53 (73.4)			CT regimens				
N1	4 (8.3)	11 (26.6)			A*C	1 (2.1)	10 (17.2)	*χ*^2^ = 11.691	**0.009**
ER/PR			*χ*^2^ = 0.492	0.483	A*C-T	12 (25)	18 (31.0)		
Positive	38 (79.2)	47 (73.4)			T/TC/TCb	16 (33.3)	26 (44.8)		
Negative	10 (20.8)	17 (26.6)			None	19 (39.6)	10 (15.6)		
M-T			*χ*^2^ = 4.511	0.211	CT cycles			*χ*^2^ = 8.434	**0.015**
A	12 (25)	7 (10.9)			0	19 (39.6)	10 (15.6)		
B	25 (52.1)	35 (54.7)			4	14 (29.2)	23 (35.9)		
Her-2(+)	5 (10.4)	9 (14.1)			>4	15 (31.2)	31 (48.5)		
Three negatives	6 (12.5)	13 (20.3)			Interval days between RT and CT	15.5 (7)	17 (9.5)	*Z* = −0.262	0.793
Her-2			*χ*^2^ = 0.771	0.38					
Positive	8 (16.7)	15 (76.6)			Tumor bed dose escalation			*χ*^2^ = 0.67	0.413
Negative	40 (83.3)	49 (10.9)							
Location			*χ*^2^ = −0.971	0.548	SIB	21 (43.8)	33 (51.6)		
Right	26 (54.2)	31 (48.4)			SB	27 (56.2)	31 (48.4)		
Left	22 (45.8)	33 (51.6)							

Sentinel LN-bio, sentinel lymph node biopsy; Armpit cleaning, axillary lymph node dissection; ER/PR, estrogen receptor/progesterone receptor; M-T, molecular type; Her-2, human epidermal growth factor receptor 2; CT, chemotherapy; A*, anthracycline; C, cyclophosphamide; T, paclitaxel; Cb, carboplatin; RT, radiotherapy; SIB, simultaneous integrated boost; SB, sequential boost.

The bolded values indicate that the figures have statistical significance (P < 0.05).

### Clinical factors associated with grade 2 or higher PLC reduction during radiotherapy

The univariate analysis of clinical factors associated with a reduction in grade 2 or higher PLC during radiotherapy, as presented in **Table 3**, indicates that receiving chemotherapy or not, the quantity of chemotherapy cycles, and initial PLC levels prior to chemotherapy and radiotherapy emerged as significant predictors (*P* < 0.05). The univariate analysis found that the differences in chemotherapy regimens had no statistical difference in the reduction of PLC (*P* > 0.05). To circumvent the issue of unstable and inaccurate coefficient estimates, the final multivariate regression analysis encompassed only those factors that were statistically significant. The initial PLC levels prior to chemotherapy constituted an independent risk factor for a reduction in PLC of at least two grades during radiotherapy (*P* = 0.027).

**Table 3:** Logistic regression analysis of factors affecting the reduction of PLC grade 2 and above in 112 breast cancer patients.

Item	Univariate logistic		Multivariate logistic	
	OR (95% CI)	*P*	OR (95% CI)	*P*
Age (years)	0.998 (0.964~1.034)	0.93		
Preservation	4.259 (0.888~20.438)	0.07		
Location	1.258 (0.594~2.664)	0.549		
Quadrant		0.267		
Quadrant (inside-up *vs*. inside-down)	0.389 (0.105~1.445)	0.158		
Quadrant (inside-up *vs*. outside-up)	0.475 (0.179~1.259)	0.134		
Quadrant (inside-up *vs*. outside-up)	1.185 (0.247~5.677)	0.832		
Histological		0.916		
Histological (I–II *vs*. III)	1.2 (0.506~2.845)	0.679		
Histological (I–II *vs*. others)	1.04 (0.389~2.781)	0.938		
T		0.252		
T (Tis *vs*. T1)	2.116 (0.631~7.102)	0.225		
T (Tis *vs*. T2)	3.2 (0.811~2.647)	0.097		
N	2.283 (0.679~7.673)	0.182		
ER/PR	0.728 (0.299~1.773)	0.484		
Her-2	1.531 (0.589~3.975)	0.382		
Molecular-T		0.23		
Molecular-T (A *vs*. B)	2.4 (0.828~6.956)	0.107		
Molecular-T (A *vs*. Her-2(+))	3.086 (0.7341~2.981)	0.124		
Molecular-T (A *vs*. three negatives)	3.714 (0.969~14.233)	0.056		
CT regimens		**0.021**		
CT regimens (AC *vs*. AC-T)	0.15 (0.017~1.329)	0.088		
CT regimens (AC *vs*. T/TC/TCb)	0.163 (0.019~1.392)	0.097		
CT regimens (AC *vs*. none)	0.053 (0.006~0.472)	**0.009**		
CT cycles		**0.018**	1.29 (0.462~3.599)	0.627
CT cycles (0 *vs*. 4)	3.121 (1.133~8.603)	**0.028**		
CT cycles (0 *vs*. >4)	3.927 (1.469~10.494)	**0.006**		
PLC (pro-CT)	0.181 (0.066~0.493)	**0.001**	0.287 (0.095~0.865)	**0.027**
PLC (lowest during CT)	0.27 (0.064~1.137)	0.074		
PLC (pro-RT)	0.092 (0.034~0.251)	<**0.001**	0.335 (0.108~0.99)	0.058
Tumor bed dose escalation (SIB *vs*. SB)	0.731 (0.344~1.55)	0.431		

The bolded values indicate that the figures have statistical significance (P < 0.05).

### The *relationship between dose p*arameters and PLC *r*eduction

As shown in **Table 4**, univariate analysis of dose factors associated with grade 2 or higher PLC reduction during radiotherapy revealed that Dmean, D40, D60, D80, and V5 of the sternum are significant predictors of grade 2 or higher PLC reduction (*P* < 0.05). Considering the issue of collinearity among the coefficients, when including D40, D80, and V5 in the multivariate analysis, it was found that the D80 of the sternum constituted an independent risk factor for a reduction in PLC of at least two grades during radiotherapy (*P* = 0.043).

**Table 4:** Logistic regression analysis of dose parameters and factors affecting the reduction of PLC grade 2 and above in 112 breast cancer patients [ $\bar{\text{x}}\pm s$ or *M* (IQR)].

Item		N/MLG (48)	M/SLG (64)	Statistic	*P*	Univariate logistic		Multivariate logistic	
						OR (95% CI)	*P*	OR (95% CI)	*P*
Breast	Volume	884.15 (424.92)	741.4 (415.1)	*Z* = −1.282	0.200	0.999 (0.998~1.001)	0.351		
	Dmean	54.17 (4.27)	53.39 (4.7)	*Z* = −1.358	0.174	0.909 (0.781~1.058)	0.217		
Sternum	Volume	48.66 ± 6.67	47.37 ± 7.6	*t* = 0.936	0.351	0.975 (0.925~1.028)	0.348		
	Dmean	7.27 (5.03)	8.76 (6.31)	*Z* = −1.931	0.053	1.12 (1.011~1.24)	0.030		
	D20	9.63 (7.03)	11.53 (10.95)	*Z* = −2.031	**0.042**	1.054 (0.999~1.113)	0.056		
	D40	6.36 (4.10)	7.52 (4.35)	*Z* = −2.287	**0.022**	1.161 (1.023~1.319)	**0.021**	0.985 (0.812~1.195)	0.879
	D60	4.89 ± 2.10	6.00 ± 2.46	*t* = −2.51	**0.014**	1.241 (1.04~1.482)	**0.017**		
	D80	2.55 ± 1.36	3.69 ± 1.98	*t* = −3.592	<**0.001**	1.489 (1.16~1.913)	**0.002**	1.517 (1.013~2.275)	**0.043**
	V5	26.51 ± 11.09	30.96 ± 8.99	*t* = −2.275	**0.025**	1.046 (1.006~1.088)	**0.024**	1.001 (0.947~1.058)	0.980
	V10	10.91 ± 7.21	12.89 ± 7.34	*t* = −1.426	0.157	1.039 (0.985~1.095)	0.157		
	V20	3.05 (5.48)	3.56 (6.99)	*Z* = −1.035	0.301	1.062 (0.968~1.165)	0.206		
	V30	1.57 (3.84)	1.75 (4.80)	*Z* = −0.935	0.350	1.086 (0.952~1.239)	0.219		
	V40	0.79 (2.01)	0.71 (2.99)	*Z* = −0.774	0.439	1.134 (0.941~1.367)	0.185		
Rib	Volume	149.42 ± 22.48	145.30 ± 20.9	*t* = 1	0.320	0.991 (0.974~1.009)	0.317		
	Dmean	13.48 ± 2.38	13.45 ± 2.69	*t* = 0.062	0.951	0.91 (0.804~1.03)	0.137		
	D20	35.50 ± 12.37	34.76 ± 13.35	t=0.297	0.767	0.996 (0.967~1.025)	0.765		
	D40	2.82 (2.03)	3.06 (2.77)	*Z* = −1.082	0.279	1.131 (0.933~1.372)	0.209		
	D60	1.05 ± 0.25	1.05 ± 0.26	*t* = −0.072	0.943	1.055 (0.245~4.537)	0.942		
	D80	0.72 (0.22)	0.73 (0.19)	*Z* = −0.735	0.462	0.241 (0.022~2.681)	0.247		
	V5	51.89 ± 11.55	52.84 ± 11.46	*t* = −0.434	0.665	1.007 (0.975~1.041)	0.662		
	V10	44.13 ± 10.45	43.71 ± 10.37	*t* = 0.208	0.836	0.996 (0.961~1.033)	0.834		
	V20	37.25 ± 9.29	36.29 ± 9.45	*t* = 0.537	0.593	0.989 (0.952~1.03)	0.589		
	V30	33.59 ± 8.99	32.13 ± 9.05	*t* = 0.846	0.399	0.982 (0.942~1.024)	0.396		
	V40	30.05 ± 8.53	28.39 ± 8.43	*t* = 1.024	0.308	0.977 (0.934~1.022)	0.306		

The bolded values indicate that the figures have statistical significance (P < 0.05).

**Figure 2:**
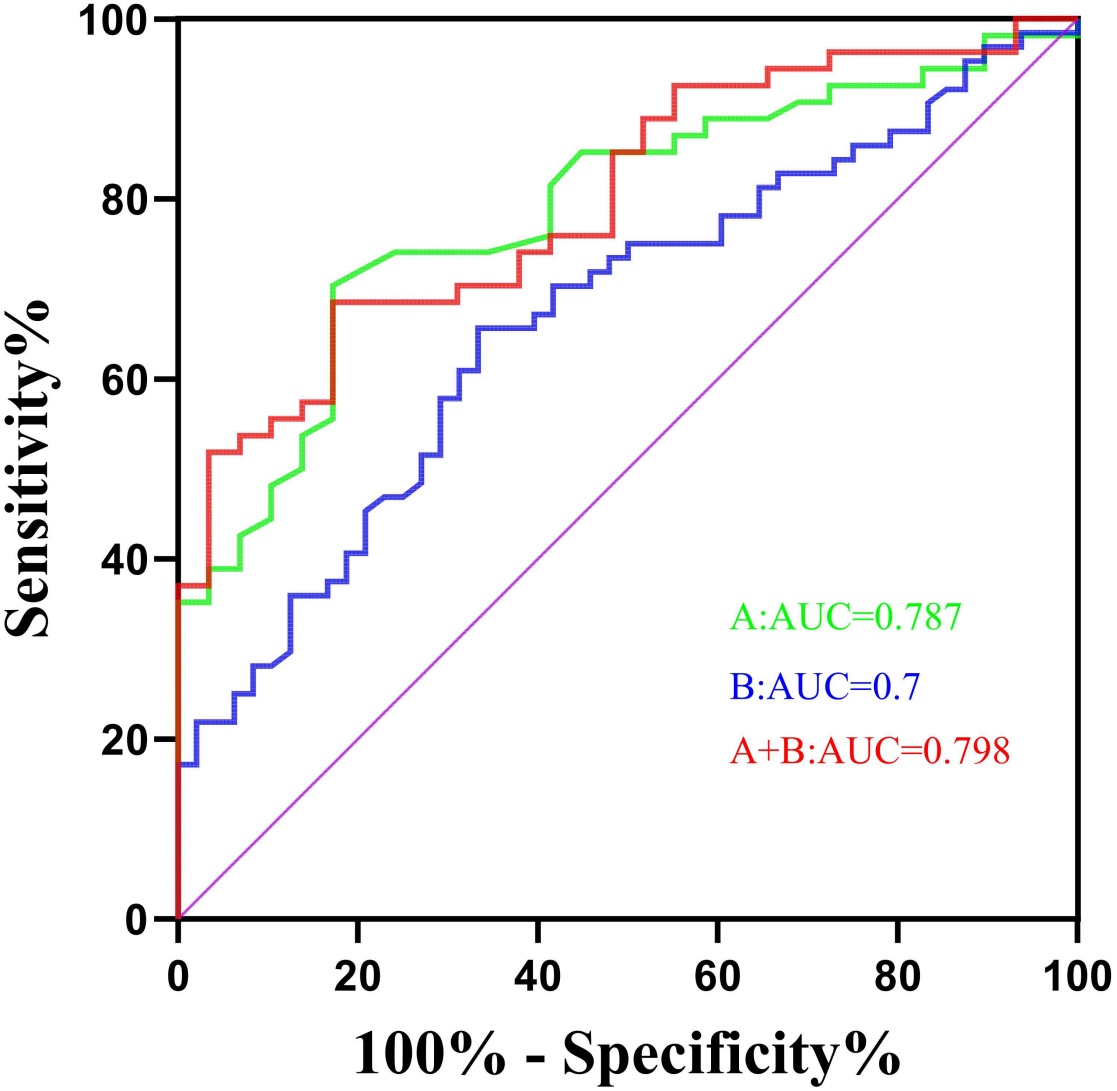
Receiver Operating Characteristic (ROC) curve.

The ROC curve was used to analyze the cutoff values of the initial PLC before chemotherapy and the D80 of the dosimetry when ≥2 grades of PLC decreased. The areas under the curve were 0.787 (*P* < 0.001) and 0.70 (*P* = 0.002), respectively. The calculated cutoff values for initial PLC and D80 process were 1.775 × 10^9^/L (sensitivity = 82.8%, specificity = 70.4%) and 2.85 Gy (sensitivity = 75.6%, specificity = 76.7%). Through the serial test, the combined AUC of the two indicators was 0.798, with a sensitivity of 80.2% and a specificity of 73.5% (**Figure 2**).

## Discussion

Blood toxicity constitutes a significant adverse effect that necessitates vigilant monitoring throughout the course of radiotherapy. Previous research efforts had predominantly concentrated on indicators including leukopenia and neutropenia; however, recent investigations have increasingly directed their focus toward lymphopenia (6–8). Lymphocytes are the most sensitive cells in the hematopoietic system to radiation, with an LD50 (the dose that reduces the survival rate of lymphocytes by 50%) of only 2 Gy (9). A single 2-Gy dose can expose 5% of circulating cells to 0.5 Gy of radiation, and after 20 to 30 fraction treatments, over 90% of circulating blood will have received at least 0.5 Gy of radiation (10). The count of lymphocytes begins to decline on the first day of radiotherapy (11). As the radiotherapy dose increased, the study established that PLC gradually decreased to its lowest point by the fourth week and began to recover from the fifth week (**Figure 1**). This is slightly different from the report by Ni WJ et al. (12), which noted a slight decrease in the fifth week of radiotherapy. The difference may be due to the fact that this study included only early-stage breast cancer patients who received sequential or synchronous tumor bed irradiation, excluding regional lymph node drainage area irradiation (above and below the clavicle), resulting in a smaller dose of cervical and thoracic spinal cord irradiation, which led to a compensatory increase in PLC from the fifth week onward.

To our knowledge, this is the first study to evaluate lymphocytopenia during conventional fractionated radiotherapy for early breast cancer patients who had undergone breast-conserving surgery. Previous studies had shown that during radiotherapy for malignant tumors of the chest, such as non-small cell lung cancer (NSCLC) and esophageal cancer, approximately 40% to 55% of patients develop grade 3 or higher lymphocytopenia (13, 14). The study revealed that 71 patients (63.39%) developed new instances of lymphopenia during radiotherapy, akin to the findings from previous studies (2, 15, 16); however, the manifestations were less severe, predominantly of grade 1–2. Only 17 patients (15.18%) experienced grade 3 lymphopenia, with no cases of grade 4 or higher adverse reactions observed. The risk of lymphopenia in this study was relatively low, possibly due to lower radiation doses to immune organs (such as the lungs and heart) and lymphoid organs (such as the cervical–thoracic spinal cord, thymus, and spleen). Therefore, the radiation-induced lymphocytopenia (RIL) in this study may occur through two mechanisms: 1) reducing circulating lymphocytes in the blood and 2) killing resident lymphocytes and progenitor cells in hematopoietic organs (such as flat bones like the sternum and ribs (17)) through irradiation.

First, lymphopenia may be related to the baseline immune status pre-radiotherapy (18). Hao (19) used the Extreme Gradient Boosting (XGBoost) algorithm to predict the risk factors for postoperative RIL in breast cancer patients, finding that baseline PLC was the most protective factor. Previous chemotherapy history also significantly affects the baseline level of PLC before radiotherapy (20, 21). This investigation, employing univariate analysis, ascertained that diminished baseline levels of PLC prior to the administration of chemotherapy and radiotherapy, exposure to chemotherapy, and the administration of four to eight cycles of chemotherapy were associated with an increased risk of experiencing a reduction in PLC of grade 2 or higher. These findings are in agreement with those reported by Yu et al. The differences in chemotherapy regimen selection do not seem to be the key factor causing moderate to severe lymphocyte reduction. However, more patients in this group choose regimens such as AC-T or TCb. Tolaney SM (22) found that severe lymphocyte reduction was associated with the concurrent use of anthracycline and taxane drugs in adjuvant therapy. However, this regimen has also been reported to cause an increase in lymphocytes in breast cancer patients (23). Currently, it is difficult for us to independently assess the impact of each chemotherapy drug on lymphocyte reduction. More studies are needed in the future to clarify the subsequent effect of chemotherapy drugs before radiotherapy on lymphocyte reduction caused by radiotherapy. At the same time, chemotherapy regimens such as AC-T and TCb often require patients to undergo more chemotherapy cycles. We speculate that whether the chemotherapy regimen indirectly affects the baseline level of lymphocytes before radiotherapy by prolonging the chemotherapy cycle, this needs to be confirmed through a prospective cohort study.

Multivariate analysis revealed that the long-term risk of radiation-related lymphocyte damage was more closely related to the baseline immune-hematopoietic system function before chemotherapy (pre-chemotherapy PLC) rather than the immediate state before radiation (pre-radiation PLC), which is different from previous reports because the factor of pre-chemotherapy PLC was not taken into account. From a biological perspective, we speculate that multiple lines of chemotherapy would severely damage lymphoid progenitor cells in the bone marrow, potentially leading to a long-term or even permanent decline in the lymphocyte regeneration capacity. Unlike neutrophils, which can rapidly recover after the chemotherapy break, the recovery of lymphocyte damage caused by chemotherapy may take several months or years. The lymphocyte count before radiation is only an instantaneous manifestation of the residual immune system after chemotherapy and cannot reflect the baseline level of lymphocyte “quality” and “regeneration potential” of the patient (24).

**Figure 3:**
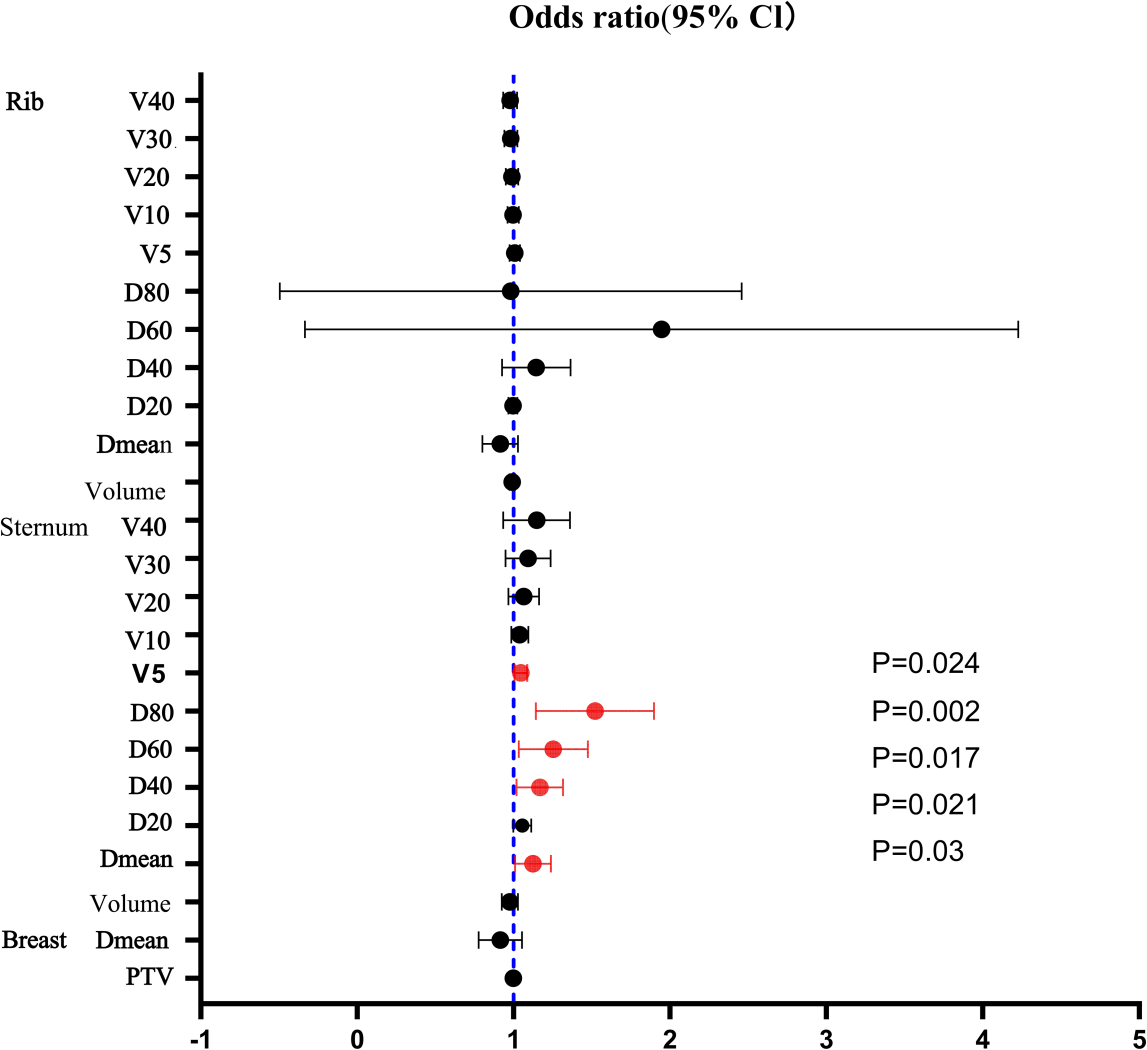
Odds ratio (95% CI).

Additionally, the reduction in PLC due to radiotherapy is influenced by the baseline level of PLC and may also be related to the irradiation of hematopoietic organs such as the sternum and ribs. Fang QQ et al. (25) reported a negative correlation between PLC reduction after radiotherapy following modified radical mastectomy for breast cancer and V5, V10, V20, V30, Dmean, D20, and D80 (*β* range: −0.370~0.245, *P* range: 0.005~0.039) of the sternum; Wang Qiang (26) collected radiotherapy dose parameters from 79 patients who underwent breast-conserving surgery and found a certain correlation between rib V5 and Dmean and grade 2 bone marrow suppression (*P* = 0.038 and 0.027, respectively); similarly, Hao (19) found from a dosimetric parameter perspective that lymphocytes are sensitive to radiation doses below 4 Gy, and the irradiation volume is more influential than the irradiation dose. This study conducted a univariate analysis of dose parameters for the entire breast, sternum, and ribs, revealing that Dmean, D40, D60, D80, and V5 of the sternum are associated with lower lymphocytopenia (**Figure 3**). Multivariate analysis showed that an increase in D80 of the sternum is an independent risk factor for grade 2 or higher PLC reduction. We have summarized the regular characteristics between the volume of the sternum exposed to radiation, the radiation dose, and the occurrence of lymphocyte depletion. Specifically, it includes the following: 1) Lymphocytes are sensitive to radiation doses above 2.85 Gy to the sternum; 2) in terms of promoting lymphocyte depletion, the effect of the volume of the sternum exposed to radiation is significantly greater than that of the radiation dose; and 3) by controlling the volume of the sternum exposed to radiation, the occurrence probability of lymphocyte depletion will increase. Therefore, when planning radiotherapy, in addition to comprehensively considering the radiation doses to the heart and lungs, it is also necessary to avoid exposing a large volume of the sternum to a lower dose of radiation. Currently, the biological mechanism by which limiting the sternum dose leads to lymphocyte reduction has not been fully elucidated. This study further highlights the issues that need to be addressed in future research.

Based on this study, it can be inferred that a PLC level lower than 1.775 × 10^9^/L before chemotherapy and a D80 value of the sternum greater than 2.85 Gy are independent risk factors for grade 2 or higher radiation-induced lymphocyte injury in postoperative adjuvant radiotherapy for early breast cancer patients. The combined prediction of these two factors can increase the AUC to 0.798. In clinical practice, it is necessary to enhance the lymphocyte reserve of patients before treatment (especially before chemotherapy), and when making radiotherapy plans, in addition to considering the radiation doses to the heart and lungs, it is also necessary to avoid the sternum as much as possible and reduce the D80 dose of the sternum to minimize the risk of moderate to severe lymphocyte reduction in patients.

In patients receiving whole-breast radiotherapy subsequent to breast-conserving surgery for early-stage breast cancer, clinical factors, notably the baseline lymphocytes prior to chemotherapy, can impact the incidence of lymphocytopenia. Reducing the sternum radiation dose can decrease the incidence of grade 2 or higher lymphocytopenia, providing valuable guidance for clinicians in delineating target areas and for physicists in planning. However, this study has several limitations: 1) This is a small-sample retrospective analysis, which may introduce selection bias and sample size limitations, affecting the results; 2) the baseline levels of the two groups differ (M/SLG received more chemotherapy cycles), which could affect the strength of the association between the sternum and rib dose parameters and the reduction in grade 2 or higher lymphocytopenia; 3) in this study, bone marrow was defined using the external contours of the sternum and ribs. When delineating the target area on CT, it was impossible to distinguish between erythroid and non-erythroid osteoblasts, inadvertently increasing the volume of bone marrow, which could affect the results. 4) Hypofractionated whole-breast radiotherapy has now become the recommended standard treatment protocol for the majority of patients with early-stage breast cancer worldwide. Sun (27) believes that reducing the number of radiotherapy sessions and shortening the total radiotherapy time may lower the risk of RIL. Moreover, Liu Yihan (28) found that compared with the conventional fractionation radiotherapy protocol, breast cancer patients receiving hypofractionated radiotherapy have a lower risk of radiation-induced lymphocyte depletion. This study only included patients who received conventional fractionation treatment, and the radiotherapy fractionation mode was single. Future prospective studies can be conducted to analyze the impact of different fractionation modes on lymphocytes. Currently, such studies are limited, and larger retrospective and prospective studies are still needed. Subsequently, multicenter and prospective studies can be conducted to verify whether it is possible to limit the radiation dose to the sternum region through updated radiotherapy techniques, adjustment of the radiotherapy position, and direction of the radiation field, in order to reduce the impact on lymphocyte counts.

## Conclusion

In summary, low absolute lymphocyte counts before chemotherapy and high sternum D80 levels during radiotherapy are contributing factors to moderate to severe lymphocyte reduction (<800 cells/μL) during radiotherapy for early breast cancer patients.

## Data Availability

The datasets presented in this study can be found in online repositories. The names of the repository/repositories and accession number(s) can be found in the article/supplementary material.

## References

[1] Zheng RS Chen R Han BF Wang SM Li L Sun KX . Cancer incidence and mortality in China, 2022. Chin J Oncol. (2024) 46:221–31. doi: 10.3760/cma.j.cn112152-20240119-00035, PMID: 38468501

[2] Sun G-Y Wang S-L Song Y-W Jin J Wang W-H Liu Y-P . Radiation-induced lymphopenia predicts poorer prognosis in patients with breast cancer: A *post hoc* analysis of a randomized controlled trial of postmastectomy hypofractionated radiation therapy. Int J Radiat Oncol Biol Phys. (2020) 108:277–85. doi: 10.1016/j.ijrobp.2020.02.633, PMID: 32147519

[3] Cho O Chun M Kim SW Jung YS Yim H . Lymphopenia as a potential predictor of ipsilateral breast tumor recurrence in early breast cancer. Anticancer Res. (2019) 39:4467–74. doi: 10.21873/anticanres.13620, PMID: 31366546

[4] Gutkin PM Kozak MM Von Eyben R Horst KC . Lymphopenia and clinical outcomes in patients with residual nodal disease after neoadjuvant chemotherapy for breast cancer. Cancer Causes Control. (2020) 31:1021–6. doi: 10.1007/s10552-020-01337-6, PMID: 32888164

[5] Offersen BV Boersma LJ Kirkove C Hol S Aznar MC Biete Sola A . ESTRO consensus guideline on target volume delineation for elective radiation therapy of early stage breast cancer. Radiother Oncol. (2015) 114:3–10. doi: 10.1016/j.radonc.2014.11.030, PMID: 25630428

[6] Damen PJJ Kroese TE Van Hillegersberg R Schuit E Peters M Verhoeff JJC . The influence of severe radiation-induced lymphopenia on overall survival in solid tumors: A systematic review and meta-analysis. Int J Radiat Oncol Biol Phys. (2021) 111:936–48. doi: 10.1016/j.ijrobp.2021.07.1695, PMID: 34329738

[7] Venkatesulu BP Mallick S Lin SH Krishnan S . A systematic review of the influence of radiation-induced lymphopenia on survival outcomes in solid tumors. Crit Rev Oncol Hematol. (2018) 123:42–51. doi: 10.1016/j.critrevonc.2018.01.003, PMID: 29482778

[8] Pike LRG Bang A Mahal BA Taylor A Krishnan M Spektor A . The impact of radiation therapy on lymphocyte count and survival in metastatic cancer patients receiving PD-1 immune checkpoint inhibitors. Int J Radiat Oncol Biol Phys. (2019) 103:142–51. doi: 10.1016/j.ijrobp.2018.09.010, PMID: 30227198

[9] Nakamura N Kusunoki Y Akiyama M . Radiosensitivity of CD4 or CD8 positive human T-lymphocytes by an *in vitro* colony formation assay. Radiat Res. (1990) 123:224. doi: 10.2307/3577549, PMID: 2117766

[10] Saito T Toya R Matsuyama T Semba A Oya N . Dosimetric predictors of treatment-related lymphopenia induced by palliative radiotherapy: Predictive ability of dose-volume parameters based on body surface contour. Radiol Oncol. (2017) 51:228–34. doi: 10.1515/raon-2016-0050, PMID: 28740459 PMC5514664

[11] Zhou ZM Xu T Shu Q . Thyroid dosimetric comparison of different techniques for supraclavicular radiotherapy of breast cancer and their effects on survival time and lymphocyte subsets. Chin J Med Phys. (2021) 38:143–7. doi: 10.3969/j.issn.1005-202X.2021.02.003

[12] Ni WJ Song LN Yang H Liu XL Zhang N Sun BJ . The influence of lymphopenia on prognosis in radiotherapy after mastectomy for breast cancer. J Mod Oncol. (2024) 32:3241–7. doi: 10.3969/j.issn.1672-4992.2024.17.012

[13] Zhao Q Chen G Ye L Shi S Du S Zeng Z . Treatment-duration is related to changes in peripheral lymphocyte counts during definitive radiotherapy for unresectable stage III NSCLC. Radiat Oncol. (2019) 14:86. doi: 10.1186/s13014-019-1287-z, PMID: 31133034 PMC6537222

[14] Deng W Xu C Liu A Van Rossum PSN Deng W Liao Z . The relationship of lymphocyte recovery and prognosis of esophageal cancer patients with severe radiation-induced lymphopenia after chemoradiation therapy. Radiother Oncol. (2019) 133:9–15. doi: 10.1016/j.radonc.2018.12.002, PMID: 30935587

[15] Chen F Yu H Zhang H Nong Y Wang Q Jing H . Risk factors for radiation induced lymphopenia in patients with breast cancer receiving adjuvant radiotherapy. Ann Transl Med. (2021) 9:1288. doi: 10.21037/atm-21-2150, PMID: 34532425 PMC8422134

[16] Kobzeva I Astrelina T Suchkova Y Malivanova T Usupzhanova D Brunchukov V . Effect of radiation therapy on composition of lymphocyte populations in patients with primary breast cancer. J Pers Med. (2023) 13:1399. doi: 10.3390/jpm13091399, PMID: 37763166 PMC10532880

[17] Hemmingsson J Svensson J Hallqvist A Smits K Johanson V Bernhardt P . Specific uptake in the bone marrow causes high absorbed red marrow doses during [^177^Lu]Lu-DOTATATE treatment. J Nucl Med. (2023) 64:1456–62. doi: 10.2967/jnumed.123.265484, PMID: 37290797 PMC10478826

[18] Dixon-Douglas J Virassamy B Clarke K Hun M Luen SJ Savas P . Sustained lymphocyte decreases after treatment for early breast cancer. NPJ Breast Cancer. (2024) 10:94. doi: 10.1038/s41523-024-00698-4, PMID: 39433772 PMC11493948

[19] Yu H Chen F Lam K-O Yang L Wang Y Jin J-Y . Potential determinants for radiation-induced lymphopenia in patients with breast cancer using interpretable machine learning approach. Front Immunol. (2022) 13:768811. doi: 10.3389/fimmu.2022.768811, PMID: 35799797 PMC9253393

[20] Chen F Ma L Wang Q Zhou M Nong Y Jing H . Chemotherapy is a risk factor of lymphopenia before adjuvant radiotherapy in breast cancer. Cancer Rep. (2022) 5:e1525. doi: 10.1002/cnr2.1525, PMID: 34390318 PMC9327667

[21] Zhou Y Liu JJ Wang XH Li N Li B Li YF . Effect of hypofractionated simultaneous integrated boost radiotherapy on immune function in patients with breast cancer. Chin J Radiat Oncol. (2024) 33:627–33. doi: 10.3760/cma.j.cn113030-20230817-00053

[22] Tolaney SM Najita J Winer EP Burstein HJ . Lymphopenia associated with adjuvant anthracycline/taxane regimens. Clin Breast Cancer. (2008) 8:352–6. doi: 10.3816/CBC.2008.n.041, PMID: 18757263

[23] Wijayahadi N Haron MR Stanslas J Yusuf Z . Changes in cellular immunity during chemotherapy for primary breast cancer with anthracycline regimens. J Chemother. (2007) 19:716–23. doi: 10.1179/joc.2007.19.6.716, PMID: 18230556

[24] Terrones-Campos C Ledergerber B Vogelius IR Specht L Helleberg M Lundgren J . Lymphocyte count kinetics, factors associated with the end-of-radiation-therapy lymphocyte count, and risk of infection in patients with solid Malignant tumors treated with curative-intent radiation therapy. Int J Radiat Oncol Biol Phys. (2019) 105:812–23. doi: 10.1016/j.ijrobp.2019.07.013, PMID: 31344435

[25] Fang QQ Guo SW Gao F . Relationship between irradiated dosimetric parameters of sternal and acute hematological toxicity in radiotherapy for breast cancer patients after modified radical mastectomy. China Cancer. (2020) 29:223–9. doi: 10.11735/j.issn.1004-0242.2020.03.A011

[26] Wang Q Qu DB Luo XH Chen HL Ye T Zhang XG . Correlation between breast cancer intensity modulated radiation therapy and bone marrow suppression. Chin J Cancer Prev Treat. (2016) 23:313–7. doi: 10.16073/j.cnki.cjcpt.2016.05.009

[27] Sun GY Wang SL . Research progress on the influencing factors and prognosis of radiation-induced lymphopenia. Chin J Radiat Oncol. (2021) 30:753–6. doi: 10.3760/cma.j.cn113030-20191111-00460

[28] Liu YH Yin HT Zhou Y Zhou C Ren HR . Effect of HFRT and CFRT on peripheral blood lymphocytes in patients with breast cancer. Chin J Radiol Health. (2022) 31:279–83. doi: 10.13491/j.issn.1004-714X.2022.03.004

